# Manipulating a Thermosalient Crystal Using Selective Deuteration

**DOI:** 10.1021/jacs.5c01140

**Published:** 2025-02-20

**Authors:** Alexander Angeloski, Pablo Galaviz, Richard A. Mole, Ross O. Piltz, Andrew M. McDonagh, Courtney Ennis, Dominique Appadoo

**Affiliations:** † Australian Nuclear Science Technology Organisation, Lucas Heights, New South Wales 2234, Australia; ‡ School of Mathematical and Physical Sciences, University of Technology Sydney, Ultimo, New South Wales 2007, Australia; § Department of Chemistry, University of Otago, Dunedin 9504, New Zealand; ∥ Australian Synchrotron, Australian Nuclear Science and Technology Organisation, Lucas Heights, New South Wales 2234, Australia

## Abstract

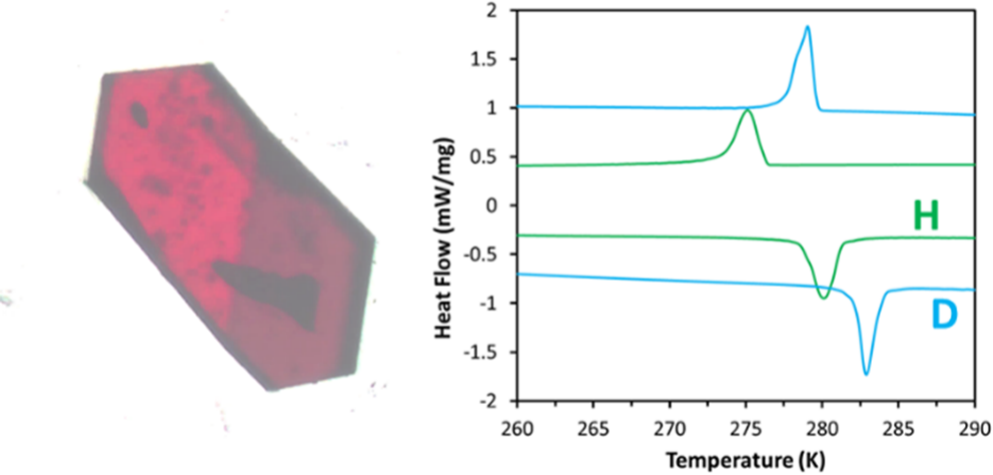

The thermosalient transformation in nickel(II) bis(diisopropyl)dithiocarbamate has been investigated using selective deuteration. The deuterated crystals undergo a reversible displacive phase transition that is ∼4 K higher in temperature compared to the protonated analogue. Neutron, synchrotron, density-functional theory, and calorimetric techniques were utilized to demonstrate the substantial effect of deuterium. All techniques demonstrated the equivalence of the mechanism on an atomic scale between the protonated and deuterated complexes. The data collected in this study reveal details of the changes of atomic motion that underpin the thermosalience inherent in this system. Deuterium decreased the frequency of atomic vibrations thus increasing the temperature of the observed transformation. This study represents a key advancement in the field of thermosalient molecular systems and provides insights into the control and manipulation of thermosalient materials.

## Introduction

Thermosalient (or “jumping”) crystals are a special example of active materials that undergo phase transformations in response to a change in temperature. First described in 1983, thermosalient materials have acquired a reputation as excellent transducers of thermal energy into kinetic energy, often manifested as motion (or “jumping”) with distances from millimeters to meters.^1−24^ The emerging field of thermosalience has caused a rapid increase in active research toward identifying and characterizing new mechanically responsive systems. Recent advances on the rise of dynamic crystals have been highlighted in several excellent reviews, undoubtedly fueling the increasing importance of thermosalient systems in the realm of active materials.^25−27^

Thermally responsive materials that can convert thermal energy into directed motion are highly desirable in industrial applications such as biomimetics, microscopic machines, energy harvesting, and actuation.^28−36^ However, such applications can be limited by material fatigue, large temperature changes for actuation, impractical temperature ranges, or other mechanical material properties. Thermosalient crystals have the potential to overcome these limitations by their ability to magnify small changes in molecular orientations into colossal macroscopic motion and extremely rapid actuation times,^14,37−43^ especially when compared to other potential actuator materials.^44−53^ Unfortunately, there are few examples of reversible thermosalient systems,^22,24,30,54,55^ with the vast majority of known thermosalient materials exhibiting irreversible, destructive transitions.^56−58^

Recently, we discovered a new reversible thermosalient system and the first reported case of a mechanically responsive dithiocarbamate complex; nickel(II) bis(diisopropyl)dithiocarbamate (NiDIPDTC)_2_.^24,59^ The transformation was unique, being reversible for at least 900 cycles and operated in environmentally accessible temperature ranges (−16 to +23 °C). The transformations were driven by atomic and molecular motion and so, we hypothesize, the properties of the thermosalient system can be manipulated by adjusting the molecular motion via structural changes.

**Figure 1:**
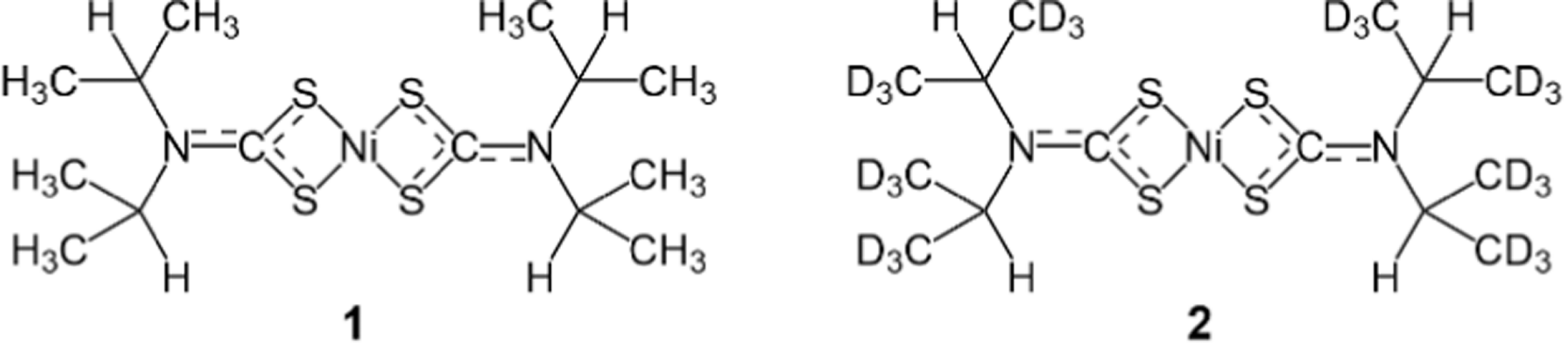
Molecular structures of the thermosalient systems examined in this work.

To test this, we selectively deuterated the sites known to be involved in the thermosalient transformation (Figure 1).^24^ Deuterium has many applications, especially when incorporated into organic molecules^60,61^ where the carbon-deuterium bonds are typically similar to those of carbon–hydrogen.

The increased atomic mass (compared to hydrogen) imparts drastic changes to the frequency of molecular vibrations,^60,62^ changing the stability and structure of crystalline polymorphs which demonstrates deuterium’s ability to manipulate crystallographic properties of materials.^63−70^ This was recently demonstrated by selective deuteration of a molecular ferroelectric,^71^ and a thermosalient.^14^ Inspired by the work of Panda et al.,^14^ we show here the first use of deuterium to manipulate the properties of a reversible mechanically responsive dithiocarbamate complex.

Fortunately, deuterium can be experimentally distinguished from hydrogen due to the reduced gyromagnetic ratio (6.53539 MHz/T for D vs 42.576 MHz/T for H), a 40× decrease in the incoherent neutron scattering cross section, and a positive coherent neutron scattering length.^72,73^ We employ a plethora of cutting-edge sophisticated techniques to probe the effect of deuterium on the structure and dynamics of the thermosalient (NiDIPDTC)_2_ system. Synchrotron and neutron techniques in conjunction with Density Functional Theory (DFT) calculations demonstrate the impact of deuterium on the thermosalient transformation. This study demonstrates an exciting use of deuterium that can be applied to other mechanical systems to alter their physical and mechanical properties.

## Results and Discussion

### Optical Characterization

**Figure 2:**
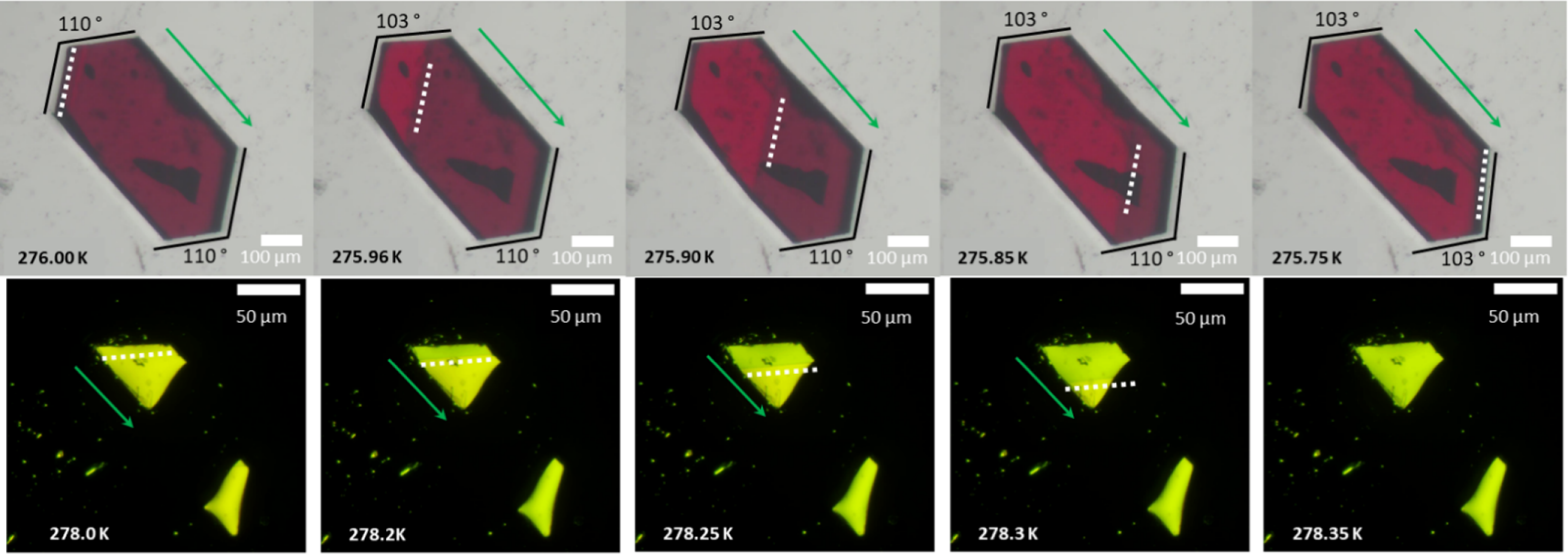
Variable temperature polarized optical microscopy of the transition front between the HT and LT phases. The optical viewing axis is aligned along the crystallographic *b*-axis of both phases. The lines of white dots indicate the approximate position and orientation of the front, while the green arrows indicate the direction of the front progression on cooling.

Figure 2 shows optical micrographs of crystals as they transition between high temperature (HT) and low temperature (LT) phases. In polarization mode, the difference in birefringence clearly distinguishes the HT and LT domains in terms of brightness while the interface between the two domains (the “thermosalient domain wall” or TDW) are seen as thin straight lines which indicate they are planar, as has been observed in other thermosalients.^74^ Thermosalient transitions are typically classified as martensitic transitions,^26^ a well-studied transition type for metallic and inorganic crystals.^75^ A planar interface between the two phases is a typical feature of martensitic transitions. Faces of the single crystal in Figure 2 were indexed (see Supporting Information) using Bravais–Friedel–Donnay–Harker (BFDH) predicted geometries. The optical direction of the micrographs are along the monoclinic axes (010) while the left–right facets are (001), and the top-bottom (100). From the intersection of the top-left and bottom-right facets we can measure the β angle of the domain, either 110 or 103° for the HT or LT phases. We index the domain wall between HT and LT domains as the (001) crystallographic plane (See Figures S1 and S2).

The speed of the TDW qualitatively increases with higher cooling or heating rates while at very high rates the crystals were observed to break. For instance, the single crystal shown in Figure 2 cracked when cooled at a rate of 80 K min^–1^ (Videos S6). At lower rates, the transformation is completely reversible with heating of the crystals producing the reverse effects to that shown in Figure 2. As a test for long-term degradation, a crystal was thermally cycled more than 900 times and showed no obvious change.^24^ We attempted to affect the motion of the domain wall through physical force applied to the crystal, though this had no apparent effect (see videos in SI, Videos S1, S2, S3, S4, and S5).

### Calorimetry

**Figure 3:**
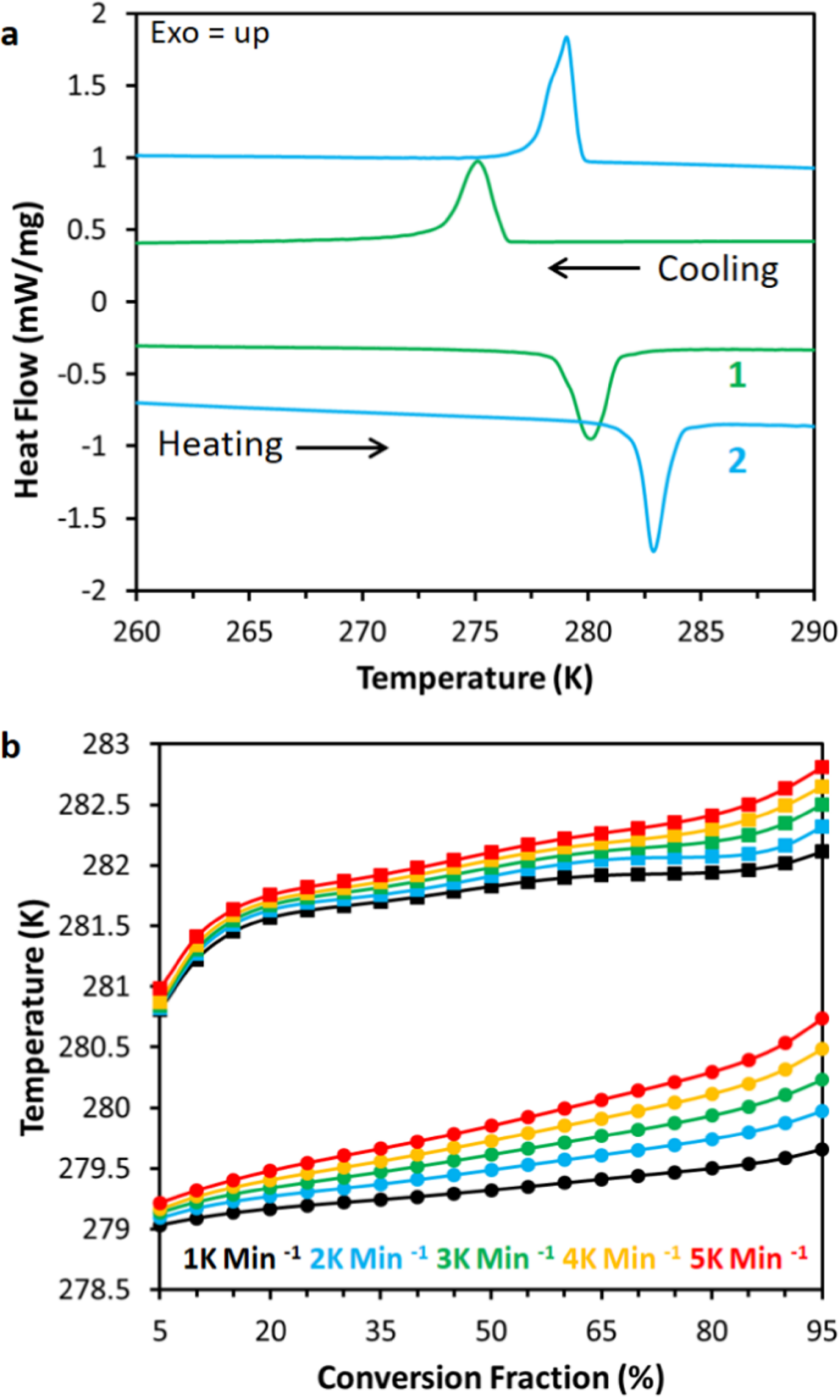
(a) Differential scanning calorimetry for **1** (green) and **2** (blue). (b) Rate dependent variance of the temperature for the LT to HT fractional conversion of **1** (circles) and **2** (squares).

Differential scanning calorimetry (DSC) data for **1** and **2** are shown in Figure 3. The transformations were reversible, with the temperatures of the transitions being affected by deuteration. Both compounds contain only a single peak during the heating and cooling processes. The exothermic peaks at ∼276 and 280 K correspond to the HT to LT phase transitions for forms of **1** and **2**, while the endothermic peaks at ∼278 and 282 K are the reverse transformations of **1** and **2**. The onsets of HT to LT and LT to HT transformations are ∼4 K higher for **2** than **1** (Figure 3a), though both complexes have the same hysteresis range of ∼2.5 K between phases. Integration of the peak areas during the HT to LT conversion yielded Δ*H* values of 2.7 kJ mol^–1^ for both **1** and **2**, indicating that mechanisms for transformation are equivalent.

Variable heating rate DSC experiments (Figure 3b) showed linear relationships between heating rate and the temperature of fractional conversion for **1** and **2**, which demonstrates the transformation is dependent only on temperature with no measurable rate dependence, in agreement with previous calorimetric studies of **1**.^24^ The partial proton content of **2** (∼18% by moles) is evident in the low conversion regime between 5 to 15% (Figure 3b); the partially deuterated isotopologues have conversion temperatures more closely approximating that of **1**, and thus the low conversion region has a slope tending toward lower temperatures. Likewise, these partially protonated isotopologues are the cause of slight asymmetry and shoulder in the calorimetry of **2** (Figure 3a). The increasing difference between the temperatures for 5 and 95% conversion with increasing rates of temperature change (Figure 3b) is a consequence of the instrument and sample thermal resistances. When looking at the low (e.g., 1 K min^–1^) rates, the difference between 5 and 95% conversion is ∼0.2 K, which represents the condition where thermal transfer from the environment to the sample is not a limiting factor. It would therefore be expected that the small sample mass and optimum thermal contact of the crystalline faces in a single crystal should produce a small (<0.2 K) temperature difference between onset and full conversion. This is confirmed by the progression of the TDW in Figure 2, which shows complete conversion in ∼0.25 K. At higher rates of temperature change, the observed transition ranges are larger (see videos and SI) and in agreement with the calorimetry.

The number (*N*) of geometrically indistinguishable orientations during the phase transformation can be interpreted from the temperature corresponding to 50% conversion, where Δ*S* = Δ*H*/*T*,^24,76−78^ yielding a value of 3 for compounds **1** and **2**. This result is consistent with the number of disordered states determined by single-crystal X-ray.^24^ The equivalent enthalpies of transformation and number of disordered sites for **1** and **2** demonstrate an identical mechanism of transformation in both complexes. Conversely, the onset of the LT to HT transition during heating is higher for D than H, attributable to the increased mass of the methyl groups. The results for **1** are in excellent agreement with previous calorimetric studies.^24^

### Single Crystal Neutron Diffraction

**Figure 4:**
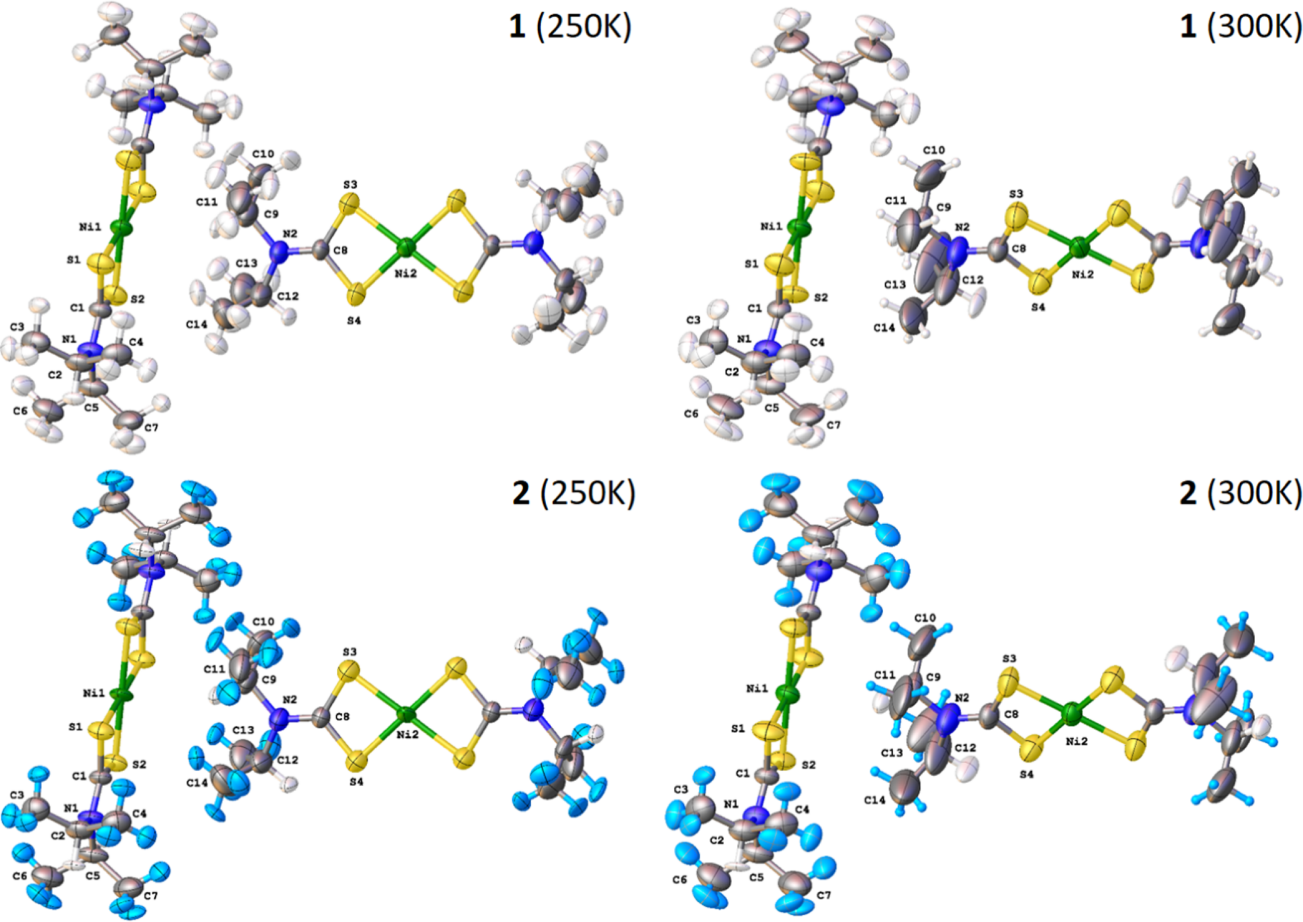
Single-Crystal neutron structures acquired on KOALA for the low-temperature and high-temperature forms of **1** and **2**. The average displacement for three disordered states is shown in the high-temperature structures for clarity.

Neutron diffraction excels at the location of hydrogen and deuterium atoms resulting in accurate distances and angles for interpreting inter- and intramolecular hydrogen-bonding interactions. Single crystal neutron structures of the high temperature and low temperature phases of **1** and **2** are displayed in Figure 4. Two crystallographically independent molecules are contained within each unit cell and are referred to as Molecule A (containing Ni1) and Molecule B (containing Ni2). There are no significant differences in the bond lengths or angles of the heavy atoms in **1** and **2** indicating that deuterium substitution has not affected the overall molecular structure. The C–D and C–H methyl bond lengths are equivalent which suggests the difference in thermosalient behavior is attributable to vibrational rather than structural effects. Likewise, the methine (C2–H2/C5-H5/C9–H9/C12-H12) bond lengths are equivalent. In the low temperature (250 K) phases, the observed thermal ellipsoids on Molecule B are significantly larger and anisotropic when compared to Molecule A indicating a greater degree of molecular flexibility on Molecule B. Indeed, Molecule B undergoes a rotation of ∼30° during the thermosalient transformation and demonstrates large disorder of the methyl groups in the high temperature structure. We were unable to resolve the disorder of these groups in the high temperature phase due to the lower limit of ∼0.89 Å wavelength neutrons used in our Laue diffraction experiments, and as such we present the disordered structures as crystallographic averages of three molecules, as previously determined by synchrotron single-crystal X-ray diffraction.^24^ The temperature-dependent increases in thermal displacement of Molecule B preceding the thermosalient transformation have been extensively investigated and reported by us elsewhere.^24^

In our previous work we have reported on the observation of 3-center-2-electron intermolecular C–H···Ni interactions in the low temperature and high temperature X-ray structures of **1**.^24,59^ C–H···metal interactions can be classified as either agostic or anagostic where the metal is d^6^ or d^8^.^59,79,80^ Agostic interactions are characterized by short H···M distances (≈1.8–2.2 Å) and C–H···M angles (90–130°)^81^ while anagostic interactions typically have longer H···M distances (≈2.3 to 3.0 Å) and larger C–H···M angles (110–170°).^82^ The neutron structures reveal C–H···Ni interactions in both forms of **1** and **2**. In the low temperature structures, the H9···Ni1 distances are equivalent at 2.967 ± 0.008 Å in **1** and 2.959 ± 0.009 Å in **2**. The C9–H9···Ni1 angles are also equivalent at 157.60 ± 0.50° for **1** and 156.20 ± 0.60° in **2**. In the high temperature structures, the H9···Ni1 distances are slightly longer in **1** (3.005 ± 0.015 Å) when compared to **2** (2.950 ± 0.030 Å). We attribute this slight difference to the disorder of the isopropyl moiety as indicated by the larger thermal ellipsoids and e.s.d for the high temperature structures. In general, the metal–hydrogen lengths are unchanged between 2.950 to 3.005 Å in all structures in agreement with the distances characteristic of anagostic interactions. The C9–H9···Ni1 angles are significantly more acute in the low temperature structures (149.50 ± 0.11° for **1** and 150.70 ± 0.16° for **2**) indicating that the C9–H9 bond will deform to maintain the C–H···Ni interaction.

The neutron structures also reveal the presence of intramolecular C–H···S interactions that are present in all known di-isopropyldithiocarbamate structures.^83^ The C12-**H12**···**S4** and C5-**H5**···**S2** interactions have equivalent (within 1.5σ) distances of ∼2.3 Å in both high and low temperature forms of **1** and **2**. Likewise, intermolecular C2-**H2**···**S4** and C5-**H5**···**S4** interactions are present at equivalent distances of ∼2.9 Å in both high and low temperature forms of **1** and **2**.

The low temperature and high temperature structures contain different intermolecular methyl–sulfur interactions due to the rotation of Molecule B relative to Molecule A as described previously.^24^ In the low temperature structure of **1**, S2···D/H-**C11** and S2···D/H-**C13** interactions are present at ∼3.2 Å. These interaction distances are unchanged (within 1.5σ) upon deuteration.

In the high temperature structures these interactions are not present and are instead replaced by S2···D/H-**C10** and S2···D/H-**C14** interactions, however, due to the disorder of Molecule B these distances are unable to be acquired with certainty. The single-crystal neutron diffraction studies clearly show the structural equivalence of **1** and **2** in both high and low temperature phases, and thus the change of the thermosalient transformation temperature is not attributable to any structural change of the system due to deuteration.

### Comparison of Diffraction Spot Images

The KOALA II neutron diffractometer was also used to compare images of individual diffraction spots from single crystals of **2** vs sample temperature. The movie “Diffraction Images” of the SI shows images containing several diffraction spots during a slow punctuated increase in sample temperature from 281.75 to 282.25 K over the course of ∼23 h. Analysis of the images, which generally contain coexisting phases within the single crystal, provides complementary information to that obtained by optical microscopy. All spots in all images can be crystallographically indexed and belong to either the LT or HT phases. The evolution of the phase fractions of both phases is shown in the SI.

For planes at increasing angle from (001) the corresponding spots of the LT and HT phases broaden and eventually split into pairs of spots. No diffuse scattering was observed between separated pairs of spots indicating high crystallinity with negligible strain within the single-phase regions of coexisting phases.

Crystallographic directions for both phases were calculated from the indexed solutions for a coexisting phase. The (010) and (001) planes are aligned within 0.06 and 0.15° respectively. When combined with the cell dimension versus temperature data,^24^ the two-dimensional (2D) lattices of the (001) planes match within 0.6% in both the *b*- and *c*-axes cell lengths, thus confirming the suitability of the (001) plane for the TDW. The observation of negligible strain within the single-phase regions confirms that any residual mismatch is strain relieved by plastic deformation and that any elastic deformation must be confined to a small volume of the crystal close to the TDW.

The evolution of the phase fraction taken from the movie images is shown in Figure S4. The growth of LT to HT starts ∼200 min after the first temperature increase. Between temperature steps the rate of change in phase fraction is generally constant and it increases with the temperature increase, as was observed using optical microscopy. However, at frames 15 and 22 the phase fraction appears to remain constant until the next temperature increase. Such behavior is typical for the pinning of a transition.^84−92^ When the TDW encounters an obstacle such as a crystal imperfection, or possibly the glue or grease used to mount the crystal, moving past such an obstacle requires a larger driving force. Once the obstacle is overcome the TDW appears to move at a constant speed dependent on the new temperature. We suggest that if the first temperature step was to 282.25 K, instead of 281.95 K, then all such obstacles would be overcome and the phase fraction would change at a constant rate of ∼0.1% per minute. For a crystal of ∼2 mm length in the 001 this corresponds to the TDW moving at ∼2 μm per minute for a temperature ∼0.3 K above the transition temperature.

From our optical microscopy observations, we found that a temperature ramp rate of 80 K min^–1^ resulted in the breakage of a crystal. How do we reconcile that a high ramp rate results in crystal fracture with the speed of the TDW depending on the driving force of the transition, that is temperature and not its rate of increase? The simplest explanation is that the TDW takes time to move through the crystal, and we are only concerned about the highest temperature reached before the thermosalient transition is complete. This is complicated by the speed of the TDW also depending on the temperature. Moreover, the hypothesis is consistent with the likely process that cause breakages, the inability to relieve strain quickly enough when the TDW is moving quickly through the crystal. The maximum rate of plastic deformation for metallic and inorganic materials is related to the number and rate of creation of dislocations.^93^ This is also probably true for molecular crystals, though the literature on such studies is sparse.^94^ We postulate that the threshold for crystal breakage is a function of the maximum speed of the TDW and the amount of strain that must be relieved due to lattice mismatch against the maximum rate of dislocation creation. If this is true it means that thermosalient transitions do not necessarily break crystals if the maximum speed of the TDW is sufficiently slow, that is the temperature is very close to the transition temperature.

### Nuclear Magnetic Resonance

**Figure 5:**
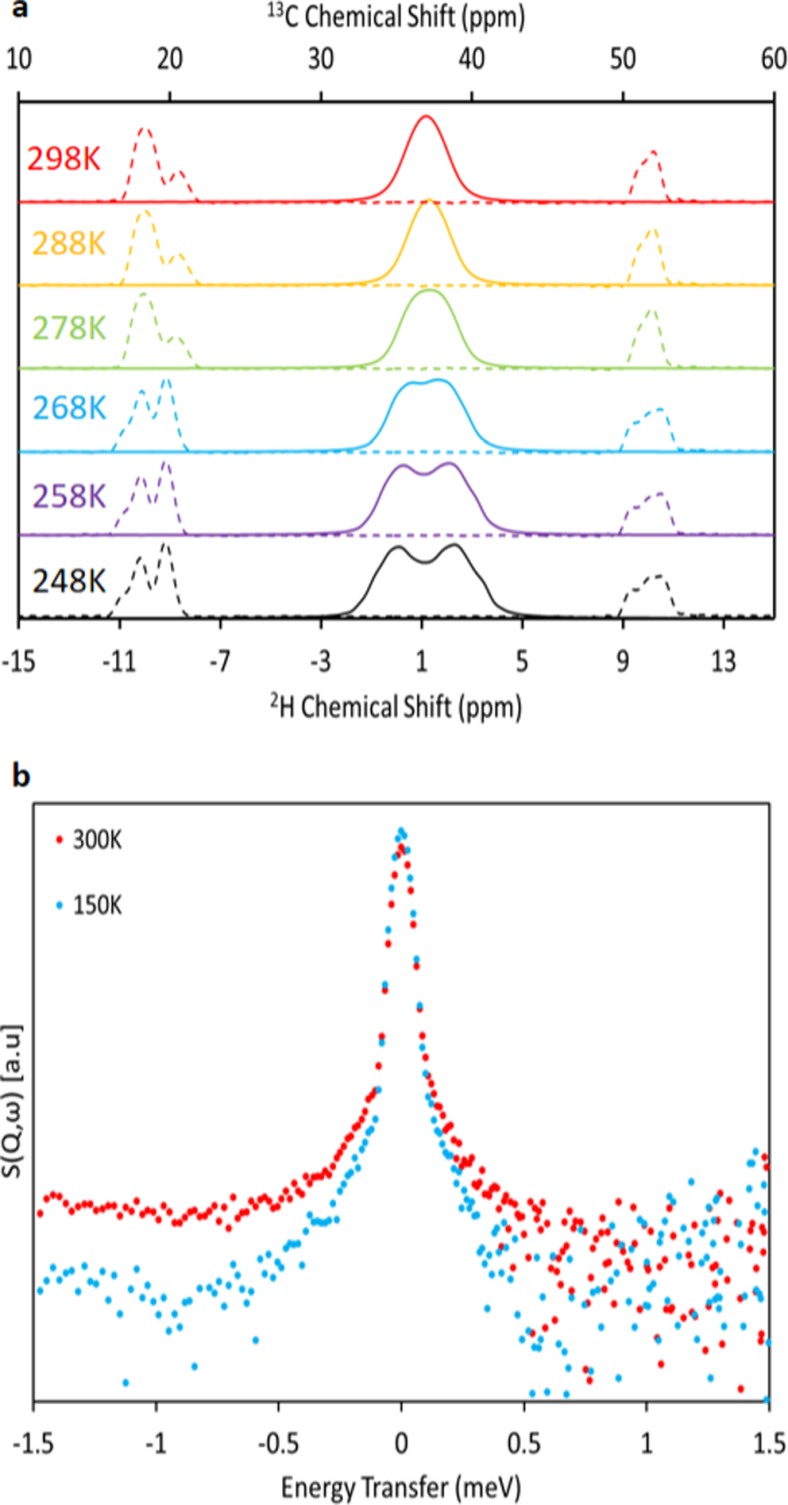
(a) Variable-Temperature ^2^H (solid lines) and ^13^C (dotted lines) solid-state NMR spectroscopy at 6 kHz MAS showing temperature dependent effects on the di-isopropyl component of **2** during cooling. The dithiocarbamato S_2_**C** moiety is omitted for clarity. (b) QENS spectra for **2** measured on PELICAN at 150 and 300 K using a neutron wavelength of 6.0 Å. The QENS signal is relatively weak due to the lower incoherent scattering of deuterium compared to hydrogen.

Variable temperature ^2^H magic angle spinning (MAS) nuclear magnetic resonance (NMR) spectra of **2** were collected over the temperature range 248 to 298 K, Figure 5a. In ^2^H NMR spectroscopy, the spectrum is dominated by the interaction of the ^2^H quadrupole moment with the electric field gradient. The width and shape of the spectral lines are therefore dependent on changes in the molecular motion^95^ which makes solid-state ^2^H NMR spectroscopy useful for obtaining information about the dynamics of a system. Indeed, solid-state NMR has been shown to be a valuable technique for the evaluation of diffusion and ion mobility in dynamic materials.^95−98^

At 248 K, two resonances are observed centered at 1 ppm which are assigned to the isopropyl −CD_3_ moieties. The lack of any additional signals clearly demonstrates the localization of deuterium on the methyl groups without any observable migration to the isopropyl −CH moiety. The observation of multiple broad deuterium resonances in the solid state is attributable to the presence of intra- and intermolecular C–D···S interactions causing an inequivalence of the isopropyl moieties.^24,59,83,99−105^ These interactions and the observation of multiple methyl/methine signals are characteristic of metal DIPDTC complexes and have been described in detail elsewhere.^83^

The temperature dependence of the deuterium spectra for **2** is shown as a continuous variation of the line shape during heating from 248 to 288 K, characteristic for an increase in the amplitudes of in-plane motion.^95,98^ Above 258 K, the line shapes are perturbed by an increase in local motion resulting in the coalescence of the multiple broad resonances at 278 K. In the temperature range where the thermosalient phase transition is observed by calorimetry, the changes in the spectra appear to show no evidence of any phase change or other significant event. The coalescence of resonances at 278 K indicates movement of the deuterium atoms occurs at a rate equivalent to the NMR time scale, where the rate of atomic motion approaches the frequency separation between the two spectral features. Upon further heating, the resonances narrow, reaching a minimum line width at 288 K indicative of thermally activated molecular displacement. The increased molecular motion above 280 K is in agreement with the single-crystal neutron structures which show a large thermal disorder in the HT structure. The observation of thermally activated molecular motion is in agreement with increased atomic vibration on heating observed in our earlier study.^24^Figure 5a also shows the result of variable temperature ^13^C MAS NMR studies performed to investigate the thermal behavior of the heavy-atoms in **2** during heating. Within the HT phase temperature regime from 278 to 298 K, there is no observation of a temperature dependent narrowing of the resonances demonstrating the relative rigidity of the carbon atoms within the isopropyl moiety when compared to the mobile deuterium atoms observed and discussed above. There are clear changes in the ^13^C resonances of the high temperature and low temperature phases such as the relative sizes of the methyl (∼18 ppm) and methine (∼53 ppm) resonances are swapped, attributed to the rotation of Molecule B. In the low-temperature structure, the narrower resonances are attributable to the increased rigidity of the low temperature phase which lacks positional disorder of the di-isopropyl groups as shown by single crystal neutron diffraction studies. There is no observation of a temperature dependent narrowing of the resonances prior to transformation.

When comparing the ^2^H and ^13^C spectra it is apparent that solid-state deuterium NMR is more sensitive to the rotation of methyl groups in the solid state causing different chemical environments in the ^2^H atoms, while ^13^C requires more drastic motion to change the local environment of C in the −CD_3_ groups and is therefore more sensitive to changes in local environments due to the phase transition.

### Quasi-Elastic Neutron Scattering

The observed temperature dependent thermal motion of the methyl moieties in **2** is reaffirmed by quasi-elastic neutron scattering (QENS) (Figure 5b). The quasi-elastic spectra obtained at 150 and 300 K clearly show a difference in the quasi-elastic broadening, where the width of the quasi-elastic contribution was narrower at low temperatures. The pronounced increase in the QENS broadening of the elastic line over the whole accessible Q range at 300 K is an unambiguous sign of dynamic disorder in the sample.^98,106^ The measured dynamic structure factor, S(**Q**,ω) contains the sum of coherent and incoherent contributions, from which under normal circumstances an elastic incoherent structure factor (EISF) can be determined. The EISF yields quantitative information about the geometry of the motions.^98,107−109^ Unfortunately, an EISF could not be obtained due to contamination of the QENS signal by Bragg reflections and low-energy acoustic vibrations, as can be seen in Figure 5(b) by the increased intensity in the *E* < −1 meV range. Nevertheless, on a qualitative level, the increased broadening of the quasi-elastic contribution to the QENS signal in the 300 K collection is attributable to an increased methyl group rotational radii, in agreement with the observed disorder in the single crystal neutron data (Figure 4), the coalescence of deuterium signals from the −CD_3_ moieties in the solid-state NMR (Figure 5a), and our previous study demonstrating the temperature dependent increases in anisotropic displacement of the −CH_3_ moieties.

### Inelastic Neutron Scattering

**Figure 6:**
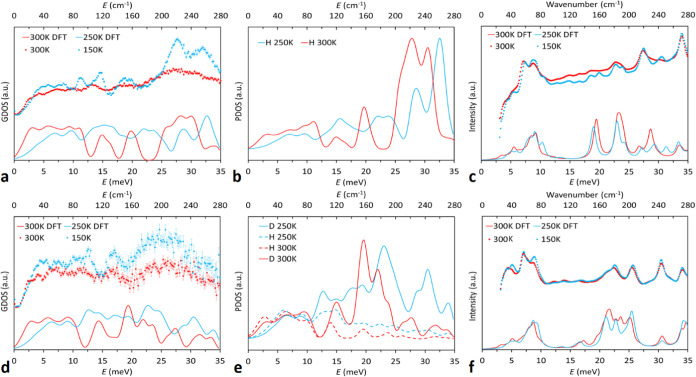
Experimental (4.69 Å neutrons) and DFT calculated INS spectra at different temperatures for **1** and **2** (a, d)*. DFT predicted partial phonon DOS showing contributions from H and D to the DOS of **1** and **2** (b, e)*. Experimental and DFT calculated synchrotron THz spectra at different temperatures for **1** and **2** (c, f). * The average of three static disordered 300 K structures was used to calculate the predicted DOS at 300 K.

Inelastic neutron scattering (INS) was used to determine the generalized phonon density of states (GDOS) in the energy range of 0–35 meV at 150 and 300 K for **1** and **2** (Figure 6). The GDOS for **1** and **2** is dominated by almost dispersionless optic branches with energies in a range between 3 to 35 meV.

Unlike Raman, IR or THz spectroscopy, which probe the Brillouin zone center (Γ or **q** = 0,0,0), neutrons are scattered by the atomic nuclei directly and thus there are no selection rules for INS spectra, allowing the observation of vibrational modes which are otherwise invisible in Raman or IR spectroscopies.^96^ Inelastic neutron scattering spectra of powdered samples collected on PELICAN (Figure 6) are considerably broadened at higher temperatures as a result of the Debye–Waller factor which is particularly noticeable in the 300 K spectra. The experimental spectra for **1** show considerably higher intensity of spectral features when compared to those for **2** which is attributed to the stronger incoherent scattering from hydrogen. The observed signals in **2** are considerably weaker than in **1** as deuterium has only 1/40 the incoherent scattering cross-section of protium.^72^ The observed increases in experimental intensities of the 150 K measurements when compared to the 300 K measurements is indicative of orientationally disordered crystals coupled with increased thermal motion.^24,110,111^

### Comparing INS to DFT Results

Comparisons of the experimentally derived INS spectra to those calculated by DFT provide information about the vibrations of the molecular groups in **1** and **2** before and after the thermosalient transformation. The DFT predicted spectra reproduces all observed features in the experimental data. The maximum discrepancies between observed and calculated frequencies are ∼1.5 meV which is within the instrument energy resolution (∼0.5 to 2 meV) at these energies.

To gain further insight regarding the identification of molecular species contributing to the generalized density of states (GDOS), DFT predicted partial density of states (PDOS) for the contribution of H and D to the GDOS are shown in Figure 6b,e. From these plots, the experimentally observed INS data for **1** is clearly dominated by the incoherent scattering of hydrogen species at energies >10 meV, while residual hydrogen which represents the dithiocarbamato moiety, dominates features observed <10 meV in **2**. The generalized density of states between 0 to 10 meV is unchanged upon deuteration (Figure 6a,d) which suggests the causative modes for these neutron annihilations are equivalent. From the PDOS plots, the <10 meV modes are clearly caused by vibrations and oscillations of the core dithiocarbamato moiety which remains chemically unchanged upon deuteration, thus explaining the equivalence of the <10 meV experimental spectra between **1** and **2**.

Each peak of the GDOS is composed of a large number of individual overlapping vibrational modes making quantitative assignment of bands difficult. Instead, the description of the molecular motions involved in the main vibrational bands of the calculated partial DOS (Figure 6b,e) was achieved by visualization of molecular vibrations at **q**(0,0,0). The vibrational animations show that modes <10 meV are dominated by collective motions of the dithiocarbamato ligand and remain equivalent in frequency between **1** and **2**, as discussed above. For modes >10 meV the motions are of the methyl groups in **1** and **2**, i.e., the specific collective motions of –CD_3_ in **2** and −CH_3_ in **1**. These visualizations are associated with the strong features in the partial density of states for hydrogen (in **1**) and for deuterium >10 meV in **2**. The changes in frequencies for –CD_3_ vs −CH_3_ were qualitatively assessed by visualization of similar molecular vibrations at **q**(0,0,0). All modes contributing to the neutron annihilations within bands between ∼11 to ∼35 meV are optically active, and in general experience a redshift of ∼2.5 to 5 meV upon deuteration. Furthermore, in both **1** and **2**, these methyl associated modes consistently redshift upon transition from the low to high temperature phases (Figure 6b,e).

### Terahertz Spectroscopy

We previously reported on the changes in IR active modes (at **q** = Γ) observed using synchrotron radiation.^24^ In Figure 6c,f we present further observations for **1** and new observations for **2**.

The low wavelength regions of the THz spectra in the sub 100 cm^–1^ range are typically attributed to driving the phase transformations of various polymorphic and mechanically responsive systems, particularly those where molecular rotations are involved.^22,112−117^ Given the observed increase in transformation temperature upon substitution of H to D, we may expect to observe differences in the <100 cm^–1^ region. Instead, the substitution of H to D does not alter the broad profile comprised of numerous lattice (translation and libration) modes residing below 100 cm^–1^; a feature that shows an increase in intensity on the blue edge at high temperature. Furthermore, modes <100 cm^–1^ capable of triggering order–disorder transitions were not observed in the visualization of atom displacements.^22,112−114^ Instead, the fundamental vibrational modes in both **1** and **2** reveal the low energy THz spectra is dominated by molecular translational and librational modes. This is in contrast to the series of intense bands located in the 120–250 cm^–1^ range for the H and D systems, where visualization of the atomic displacements confirm these modes are predominantly methyl torsion in character, with those at lower frequency coupled to out-of-plane deformation of the NiS_4_ moiety. We previously identified two near-degenerate vibrational modes at ∼182 cm^–1^ in **1** which met the criteria of being “gateway modes”;^24^ as these modes are methyl torsion in character, they experience a slight shift in their frequency upon deuteration (Figure 6c,f).

### Comparing Terahertz Spectroscopy to DFT Results

Frequencies for the DFT calculated peaks (fitted with 10 cm^–1^ Gaussian profiles in the simulated spectra) are well-aligned with experimental peak positions, although the relative intensities of the DFT predicted bands deviate from that measured. As excited torsional levels are populated at higher temperature, the corresponding bands in the THz spectra broaden and are less resolved at 300 K (most clearly observed for **2** in Figure 6f). However, as thermally populated excited states are not described by the theory (performed at 0 K), the 300 K DFT spectra instead shows torsional bands of increased frequency and intensity; as calculated for the altered NiDIPDTC coordinates and cell parameters after phase transition. It can therefore be surmised from the THz spectra and the DFT modeling that partitioning of energy into methyl torsion excited states can contribute to the dynamics of the LT to HT phase transition.

### DFT and Dynamical Instabilities

**Figure 7:**
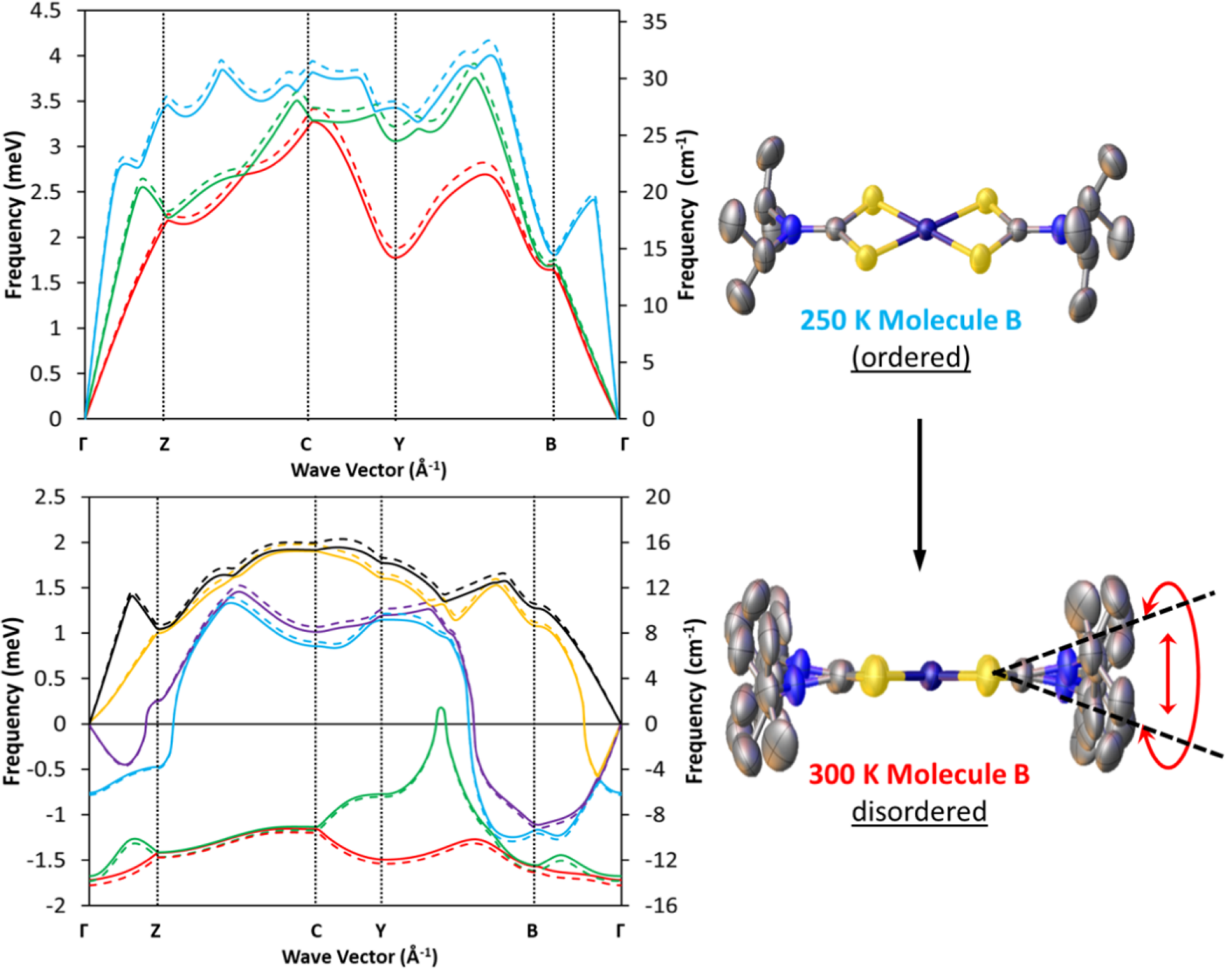
Low frequency calculated phonon dispersion curves for **1** (dashed) and **2** (solid) at 250 K (upper) and 300 K (lower). The 300 K structure of molecule B is observed experimentally as a crystallographic average of three distinct disordered fragments with large-amplitude local distortions of the isopropyl moieties. Multiple imaginary modes in the 300 K structure are predicted at the Γ point which correspond to motion of Molecule B. The schematic shows the molecular structures of the disordered molecule before and after the thermosalient transformation.

Information on acoustic-phonons was extracted by analysis of the phonon dispersion in the low-energy <3 meV regime. In Figure 7, each curve represents the frequency of a single acoustic vibrational mode as observed at each Brillouin zone within the crystal. Our experimental data from Figure 6 shows very little difference in the observed intensities of low energy transfer modes when comparing **1** and **2**. We also discussed earlier that the QENS signal was contaminated by Bragg reflections and low-energy acoustic vibrations, which in turn indicates the low energy region of the INS will be dominated by coherent scattering. These low frequency density of states between 0 and 10 meV represent flat optic phonon modes for the central dithiocarbamate NCS_2_Ni backbone, which is chemically unchanged upon deuteration and hence the dispersions of low frequency phonons also remain unchanged upon deuteration (Figure 7).

The phonon dispersions for the 300 K structures display several imaginary frequency phonon modes, as shown by the presence of negative energy dispersions. Such modes indicate an energy decrease for an atom displaced from its equilibrium positions. This paradoxical statement results from our method of phonon calculation that assumes a quadratic (harmonic) dependence of energy versus displacements. It is well-known that imaginary modes obtained from a harmonic model are predictors of dynamic instability resulting from the neglected anharmonic energy terms.^118−121^ We posit that the observation of these imaginary modes in our study are not an artifact of poor convergence of the DFT calculation. To check for this possibility, DFT calculations on the single-cell structures used as inputs for the finite displacement method were repeated using variable cell relaxation, increased force and electron energy tolerances, and different pseudopotentials. The results all produced similar dispersions with imaginary modes. Replication of the finite displacement method using each conformation of the disordered molecule B produced variations of dispersions of the imaginary modes shown in Figure 7, indicating their presence is associated with the observed experimental disorder, a dynamic instability. Visualization of the imaginary modes at the zone center (**q** = Γ) are given in the SI and show the motion of isopropyl moieties in molecule B. When these modes are interpreted alongside the findings from QENS measurements discussed above, variable-temperature single-crystal synchrotron X-ray diffraction,^24^ and variable temperature solid-state deuterium NMR, we conclude that the observation of imaginary modes is a real representation of dynamic instability in the high temperature phase. In other dynamic systems, imaginary modes are an established characteristic of materials that undergo displacive phase transitions.^118,122−130^ It is interesting to note that the imaginary modes are deepest along the Brillouin direction **B** of Figure 7 which is perpendicular to the (001) crystallographic plane, the same plane as the thermosalient domain wall of Figure 2. It is possible that this is relating to mode softening of a transverse mode which precedes a static shear of the planes, as often seen in martensitic transitions.^131^ For the 250 K structures, the phonon dispersions are more typical, where the low frequency region of the spectrum is dominated by acoustic phonons, and no dynamic instabilities in the form of imaginary frequency modes are predicted.

### Vibrational Mechanism for the Thermosalient Transformation

To explain the apparent stability of the HT structure despite the imaginary modes, we propose that the disorder of Molecule B effectively reduces the long-range coherence of the crystal which in turn dampens the long-range acoustic phonons. This dampening also applies to the imaginary modes, that is the dynamic instability that drives the displacive phase transition. The disordered fragments of Molecule B, by oscillating between three positions, are thus inhibiting the otherwise energetically favorable movement of atoms from their equilibrium positions.

On cooling to the LT structure, the reduced motion of the fragments of Molecule B eventually prevent the hopping between multiple sites and the molecule becomes trapped in a single orientation. The increase in long-range coherence removes the dampening of the zone center imaginary modes and the structure changes from dynamically to statically unstable resulting in a displacive transition where the structure relaxes to a lower energy configuration.

The reverse is true upon heating, the low-energy acoustic modes are overcome by strong localized modes resulting from the disorder and the structure relaxes to the lowest energy configuration consistent with a disordered fragment.

Regarding the effect of deuterium on the thermosalient temperature, as the dispersions in these low energy neutron annihilation regions are virtually unaffected by deuteration, we can conclude that the increased stability of the LT phase in **2** is not attributed to a direct change in acoustic behavior. Rather, the decreased vibrational amplitude of Molecule B, due to the increased mass of deuterium, reduces the dampening effect on the dynamic instabilities in the HT phase thus stabilizing the LT phase to higher temperatures. In summary, thermosalience in this type of material is a phonon driven transformation. We conclude that rotational motions of the disordered Molecule B in the high temperature phase is causative of the thermosalient transformation, reinforcing our previous claims.^24^

## Conclusions

In this study we have continued our previous investigations on the thermosalient transformation in nickel(II) bis(diisopropyl)dithiocarbamate using selective deuteration. The deuterated crystals undergo a reversible displacive phase transition that is ∼4 K higher in temperature compared to the protonated analogue. The structures of the protonated and deuterated compounds were determined to be equivalent using single-crystal neutron diffraction. Variable temperature solid-state deuterium nuclear magnetic resonance experiments revealed temperature dependent changes in the atomic displacement of deuterium atoms preceding the transformation. Quasi-elastic neutron scattering demonstrated clear differences in methyl group rotational radii between the high and low temperature phases. Density functional theory reproduced experimental inelastic neutron scattering and synchrotron terahertz absorbance spectra to reveal the atomic vibrational modes causing thermosalience. All techniques demonstrated the equivalence of the mechanism on an atomic scale between the protonated and deuterated complexes.

We show that the thermosalient transition, in our case, is an unusual combination of order–disorder and displacive mechanisms. The disorder in the high temperature phase results in strong localized optical modes that suppress long-range acoustic lattice modes which would otherwise render the phase energetically unstable to a displacive transition. Deuteration of the methyl groups decreases the strength of these optical modes thus stabilizing the low temperature phase to higher temperatures.

As acoustic phonons have zero energy at **q** = Γ, these modes, and by extension any change in their frequency or intensity are not visible using traditional techniques that probe the Brillouin zone center **q**(0,0,0) such as THz spectroscopy, FTIR, and Raman. Unsurprisingly, this is the reason we did not observe these phenomena in our previous investigation, or in the THz discussed earlier.^24^

Polarized optical microscopy combined with a comparison of neutron diffraction spot images confirms that the transition between high and low temperature phases occurs on a planar interface with little lattice mismatch between both phases. Any residual mismatch is then relieved by plastic deformation. Such behavior is also typical for martensitic transitions in metallic and inorganic systems. On the basis of these experimental technique, we also hypothesize on the requirements for reversibility of the thermosalient transition.

Overall, our study demonstrates the use of deuterium for changing the temperature of the thermosalient transformation thus allowing new insight into the mechanisms of this system; an advancement that we believe will be applicable to other thermosalient and mechanically responsive systems.

## Experimental Section

### General

Reagents were sourced from Sigma-Aldrich and used without further purification. Milli-Q water was used for all syntheses. The deuterium enrichment levels were calculated using DGet!.^62^ Computational calculations and image processing were performed using a personal computer owned by the corresponding author (dual AMD EPYC 8B12 processors for a total of 256 processing cores with 1.1 TB of RAM), and on the Setonix and GADI Supercomputers.^132^ BFDH predicted morphologies were calculated using KrystalShaper.^133^

### Synthesis

**Scheme 1:**
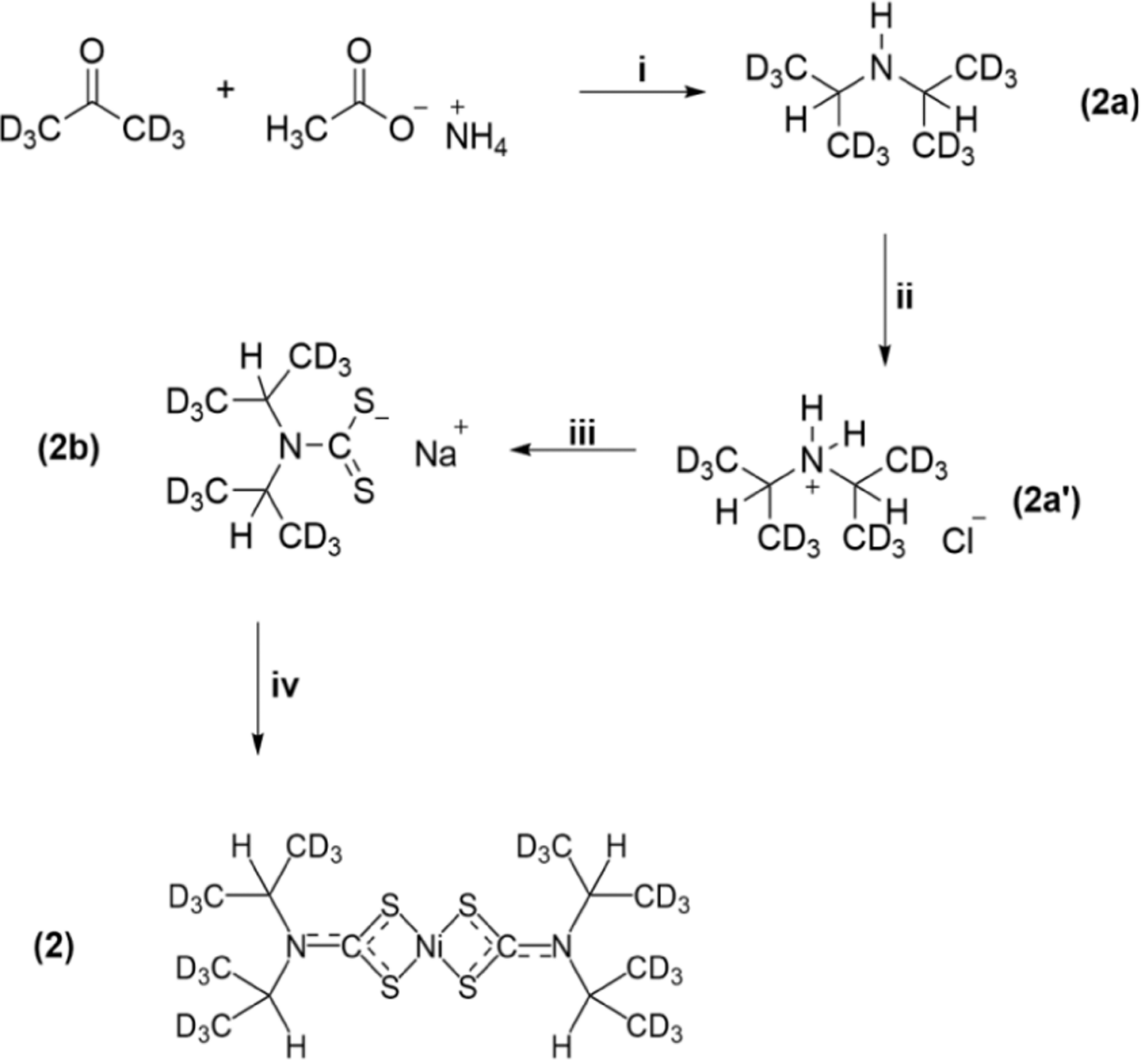
Synthesis of **2** (i) NaBH_3_CN, MeOH, 298 K, 75 Hr. (ii) 3M HCl in MeOH. (iii) Aqueous sodium hydroxide, carbon disulfide ether solution, 278 K. (iv) aqueous nickel(II) sulfate heptahydrate.

Bis(*N*,*N*-di-isopropyldithiocarbamato-S,S′)-nickel(II), **1**, was synthesized as previously described.^59,134^ Bis(*N*,*N*-di-isopropyldithiocarbamato-S,S′)-nickel(II)-*d*24 (**2)** was synthesized as shown in Scheme 1. First, 8.23 g of NaBH_3_CN (*n* = 0.1309) is dissolved in 120 mL of anhydrous methanol, to which 9.0 g of ammonium acetate is dissolved (*n* = 0.1168). The solution is stirred for 5 min, after which point 15.7 g of acetone-*d*_6_ (*n* = 0.2448, 99.5% D) is added dropwise through a condenser. A vigorous reaction occurs leading to self-reflux, and the mixture is brought to room temperature using ice, after which point the solution is stirred at room temperature for 75 h. The reaction mixture was quenched by suspension of anhydrous potassium carbonate, and the volatiles were removed using short-pass vacuum distillation. The fractions containing **2a** were collected as an azeotrope with a boiling point of 341 K. To rectify the azeotrope, 40 mL of 3 M HCl in anhydrous methanol was added dropwise, and all volatiles were removed *in vacuo*. The resultant fine-white powder **2a**′ was freeze-dried to produce 12.14 g of **2a**′ (70% yield, 84% D), which was subsequently stored under Argon. The ligand, **2b**, as a pentahydrate, was synthesized by modification of our previous methods^99^ as follows: An aqueous solution of sodium hydroxide (7.1 g in 13 mL of Milli-Q water) was cooled to 5 °C, to which 6.0 g of **2a**′ (*n* = 0.0402) was added while maintaining the solution at 5 °C. Next, an excess of carbon disulfide in 20 mL of diethyl ether was added dropwise while not allowing the mixture to exceed 5 °C. The mixture was stirred for 10 min, after which point a white slurry had formed. The slurry was dried, and a mixture of methanol/diethyl ether was added, at which point a slimy precipitate was formed. The organic layers were removed and reduced *in vacuo* until a viscous oil had formed. Dry diethyl ether was added to this oil, and crystals formed, which were filtered and washed several times with diethyl ether. The filter cake was dried *in vacuo* to produce 5.3 g of **2b** (66% yield, 84% D). Finally, **2** was prepared by reaction of aqueous **2b** (*n* = 0.0099) with aqueous nickel(II) sulfate pentahydrate (*n* = 0.00369) to produce a brilliant green precipitate which was washed with water, dissolved in chloroform, and dried over anhydrous magnesium sulfate to produce a dry solution of **2b**. The volatiles were removed *in vacuo* to yield 0.58 g of **2** (38% yield, 84% D)

### Nuclear Magnetic Resonance Spectroscopy

Variable temperature solid-state ^13^C and ^2^H nuclear magnetic spectroscopy was performed using a Bruker Avance III spectrometer. Single crystals of **2** were loosely crushed and packed in 4 mm zirconia rotors and sealed using a vespel lid. The samples, in their sealed rotors, were spun at the magic angle with a rotation rate of 6 kHz. The spectrometer frequency was set to 61.43 MHz for ^2^H and 100.63 MHz for ^13^C. The ^13^C signals were automatically frequency calibrated relative to TMS, and the ^2^H signals were manually referenced relative to their equivalent frequency in solution-state measurements.

### Differential Scanning Calorimetry

Differential scanning calorimetry (DSC) data were obtained using a Netzsch DSC 300 Caliris instrument. The instrument was calibrated before use for heat flow using an indium standard and a sapphire disc for the cell constant. Thermograms were acquired using heating rates of 0.5 to 5.0 K min^–1^, increasing in 0.5 K increments. As the thermosalient transformation is reversible, a single sample was used for all 10 measurements. The resultant thermograms were baseline-corrected (Figure S3) and converted to conversion plots (α vs K) via stepwise integration as follows: The conversion fraction (α) is obtained by stepwise integration of the endothermic peak resulting from the LT to HT transformation, whereby 100% phase conversion is defined as the total endotherm area. The temperature corresponding to partial integrals (i.e., values between 5 and 95%) for each value of α is obtained, and evaluation of kinetic parameters is performed on the resultant conversion plots. All nonisothermal kinetic analysis was performed using methods recommended by the ICTAC Kinetics Committee.^135^

### Neutron Diffraction

The structures of **1** and **2** at 250 and 300 K were investigated using single-crystal neutron Laue diffraction. The unit-cells employed were as for the X-ray determinations from our previous investigations using Synchrotron radiation and recovered from the Cambridge Crystallographic Data Centre (2072088 and 2072090) as these cannot be reliably determined using Laue neutron diffraction. Single crystals of **1** and **2** were grown from gentle evaporation of dilute chloroform solutions over a period of 9 days. Single crystal neutron Laue diffraction data were collected on the KOALA,^136^ and it is replacement KOALA II instrument which stands at an end guide position of TG3- a supermirror thermal neutron guide at the OPAL nuclear reactor at ANSTO. Single crystals were mounted on the phi axis of the KOALA diffractometer and were immobilized by adhesion to a 150 μm aluminum sheet using PTFE grease. The crystal, grease, and aluminum holder were wrapped with PTFE tape. The temperature was controlled using an Oxford Cryosystems Cobra Plus sample cryostat. Data reductions were performed using the LAUEG suite.^137,138^ The structures were refined using SHELXL^139^ with the program OLEX2.^140^ The chemical occupancies for D atoms in **2** were freely refined and converged with partial occupancies in agreement with the known overall deuterium enrichment determined using mass spectrometry.

Neutron Laue data for **1** was collected from a single-crystal (∼2 × 3 × 3.5 mm^3^). At 300 K, a total of 17 Laue diffraction images were collected in a single run (7200 s exposures) with 14° rotation of the crystal around the phi axis occurring between exposures. A total of 19537 reflections from neutrons of wavelengths between 0.850 and 1.70 Å, covering the full sphere of reciprocal space to a maximum resolution of 1.11 Å were reduced [L4R(int) = 5.0 (5.8) for 4σ observations] to yield 2193 independent reflections; 997 with *I* > 4σ(I). At 250 K, a total of 18 Laue diffraction images were collected in a single run (7200 s exposures) with 14° rotation of the crystal around the phi axis occurring between exposures. A total of 21,425 reflections from neutrons of wavelengths between 0.850 and 1.70 Å, covering the full sphere of reciprocal space to a maximum resolution of 1.11 Å were reduced [L4R(int) = 5.2 (5.7) for 4σ observations] to yield 2235 independent reflection; 1127 with *I* > 4σ(I).

Neutron Laue data for **2** was collected from a single-crystal (∼1 × 2 × 3 mm^3^). At 300 K, a total of 14 Laue diffraction images were collected in a single run (3600 s exposures) with 14° rotation of the crystal around the phi axis occurring between exposures. A total of 15,714 reflections from neutrons of wavelengths between 0.850 and 1.70 Å, covering the full sphere of reciprocal space to a maximum resolution of 1.11 Å were reduced [L4R(int) = 4.7 (4.5) for 4σ observations] to yield 2079 independent reflections; 1050 with *I* > 4σ(I). At 250 K, a total of 14 Laue diffraction images were collected in a single run (3600 s exposures) with 14° rotation of the crystal around the phi axis occurring between exposures. A total of 16,212 reflections from neutrons of wavelengths between 0.850 and 1.70 Å, covering the full sphere of reciprocal space to a maximum resolution of 1.11 Å were reduced [L4R(int) = 4.2 (3.9) to yield 2145 independent reflections]; 1163 with *I* > 4σ(I).

### Neutron Scattering

Inelastic and quasi-elastic neutron scattering (INS and QENS) data were collected at 150 and 300 K using the cold neutron time-of-flight spectrometer, PELICAN at the ACNS ANSTO.^141,142^ The instrument was configured for incident neutron wavelengths of 4.69 Å (INS) and 6.0 Å (QENS), which correspond to incident energies of 3.7 and 2.3 meV. The neutrons were scattered by the sample and detected using the 1 m high detector bank that spans 125°. Single crystals of **1** and **2**, from the same batch used to acquire single-crystal neutron structures, were sacrificed in a pestle and mortar to yield fine free-flowing powders which were packed within annular aluminum cans with a total sample thickness of 1.0 mm (corresponding to an annular spacing of 0.5 mm). A vanadium standard was measured for detector normalization and to determine the resolution function for the QENS analysis, and the background was corrected by subtraction using an empty aluminum reference can. For INS measurements, a neutron wavelength of 4.69 Å was used with a chopper frequency of 200 Hz to yield an energy resolution of Δ0.2 to Δ2 meV in energy transfer ranges between −1 to −35 meV. The corrected time-of-flight spectra were then converted to S(Q,ω). The generalized density of states (GDOS) is obtained from S(Q,ω) using the eq 1 followed by integration over Q (2)
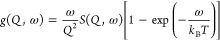
1
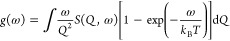
2

For QENS measurements, the instrument was optimized for 6.0 Å incident neutrons, affording an energy resolution of 65 μeV at the elastic line. All data reduction and processing was performed using the Large Array Manipulation Program (LAMP).^143^

### Variable Temperature Optical Microscopy

Polarized transmission photomicroscopy of the thermosalient transformations was performed using a modified Nikon Eclipse TE200 microscope and photomicrographs were acquired using an Amscope 18 MP-HS USB3.0 Camera. Single crystals, or fragments of single crystals, were placed atop a 0.1 mm thick glass wafer within a custom sample environment manufactured by the corresponding author. The enclosure is described briefly; a Linkam THMS600 stage was modified to accept a Peltier module. The sample temperature was modulated using a Peltier element, the hot side of which was liquid-cooled using a closed-loop liquid recirculator and controlled with a Meerstetter TEC-1161. The glass wafer was thermally bonded to the Peltier cold side, and the entire cold-assembly was entombed with a nickel-plated copper hat with a 5 mm aperture. The temperature of the sample was measured by a Class B Pt100 RTD thermally bonded to the glass substrate, and the auxiliary temperature of the nickel plated copper hat was monitored by a Class B Pt100 RTD thermally bonded to the nickel-plated copper. The sample environment was purged by a flow of dry air with a dew point of <200 K at 300 mL min^–1^. Where necessary, videos and images were compressed or converted using ffmpeg.^144^ All images were processed using Fiji.^145^

### Synchrotron Terahertz Spectoscopy

Far-IR spectroscopy was performed at the Terahertz and Far-Infrared beamline at the Australian Synchrotron. A closed loop pulsed tube cryostat (Cryo Industries) was installed on the beamlines’ Bruker IFS125 FTIR spectrometer. Single crystals of **1** and **2** were crushed to produce a fine powder, of which ∼1 mg was homogenized in paraffin wax and pressed into a pellet of 3 mm diameter. The measurements were collected under a partial helium atmosphere between room temperature and 3 K. The 20–300 cm^–1^ spectral region was accessed using the synchrotron edge-radiation source, a 75 μm Mylar beam splitter, and a liquid helium cooled silicon bolometer detector. Spectra were recorded at 4 cm^–1^ resolution, and processed using a custom python script developed by the corresponding author. Processed spectra were corrected and baselines from wax subtracted using Spectragryph software.

### Computational Details

To simulate the synchrotron THz spectra, the periodic DFT code CRYSTAL17 (at B3LYP-D3/6-311G(d) level of theory) was employed, as used earlier for simulations of **1**(24) data. The H atom coordinates for the methyl groups were labeled as D, and the frequencies recalculated at both temperatures to simulate THz spectra for **2**.

All other DFT calculations were performed using Quantum-Espresso (QE).^146−151^ Initial atomic positions from single crystal X-ray diffraction structures (CCDC 2072088 and 2072090) were used to generate input files for QE. Structures were selected at 300 and 250 K to match experimentally acquired single-crystal neutron structures presented here. The files were generated using the Materials Cloud platform.^152^ We performed an energy cutoff test (ecutwfc parameter in QE) with energies between 40 and 80 Ry. We selected 40 Ry as the optimal value with less than 0.001% relative change to the calculated energy at 80 Ry. Similarly, we performed a k-point convergence test, choosing a 2 × 2 × 2 mesh without offset. We used those parameters for relaxation and phonon calculations. We performed variable cell relaxation using damped (quick-min Verlet) dynamics, a 0.0001 force convergence threshold (forc conv thr) and a 0.002 energy convergence threshold (etot conv thr), however this produced unrealistic lattice constants We chose to use position relaxation with fixed lattice constants, VdW dispersion correction, a 1 × 10^–6^ force convergence threshold (force conv thr) and a 0.0002 energy convergence threshold (etot conv thr) for the energy minimization.

For phonon calculation, we employed the finite displacement method.^153,154^ We generated the displacement structures using Phonopy.^155,156^ Postprocessing analysis was done using Phonopy and Euphonic software.^157^

Since the unit cell contains 196 atoms, using a large supercell for phonon calculation was unfeasible. Instead, we generated 294 displacements on a 2 × 1 × 1 supercell with 392 atoms per structure. The input coordinates for the 211 supercell were those that were obtained using single-cell atomic position relaxation as described above. The resulting supercell are approximately cubic. The density of states was calculated from 0 to 35 meV with a 0.5 meV resolution and 1.4 meV Gaussian broadening width. With these parameters, each phonon calculation took ∼700 h of continuous computation using 256 processing cores, for a total computation time of ∼5000 h

## Supporting Information Available

BFDH predicted morphologies, descriptions of videos, neutron diffraction spot and calorimetric data (PDF)
Crystal Movements (Video S1) (AVI)
Thermosalient Domain Wall 1 (Video S2) (AVI)
Thermosalient Domain Wall 2 (Video S3) (AVI)
Thermosalient Domain Wall 3 (Video S4) (AVI)
Thermosalient Domain Wall 4 (Video S5) (AVI)
Crack (Video S6) (AVI)
Isothermal (Video S7) (AVI)
Imaginary Mode 1 (Video S8) (AVI)
Imaginary Mode 2 (Video S9) (AVI)
Imaginary Mode 3 (Video S10) (AVI)
Diffraction Images (Video S11) (AVI)

## Supplementary Material

ja5c01140_si_001.pdf

ja5c01140_si_002.avi

ja5c01140_si_003.avi

ja5c01140_si_004.avi

ja5c01140_si_005.avi

ja5c01140_si_006.avi

ja5c01140_si_007.avi

ja5c01140_si_008.avi

ja5c01140_si_009.avi

ja5c01140_si_010.avi

ja5c01140_si_011.avi

ja5c01140_si_012.avi
