# Supplementary material for: Manipulating a Thermosalient Crystal Using Selective Deuteration
Source: J Am Chem Soc. 2025 Feb 20;147(9):8032–47. doi: 10.1021/jacs.5c01140 (PMC11887453; doi:10.1021/jacs.5c01140)
Supplement: Supplementary file 1 — ja5c01140_si_001.pdf [file ja5c01140_si_001.pdf]

Manipulating a Thermosalient Crystal using Selective Deuteration

Alexander Angeloski[*a,b], Pablo Galaviz[a], Richard A. Mole[a], Ross O. Piltz[a], Andrew M. McDonagh[b], Courtney Ennis[c], and Dominique Appadoo[d]

[a] Australian Nuclear Science Technology Organisation, New South Wales 2234, Australia.

[b] School of Mathematical and Physical Sciences, University of Technology Sydney, New South Wales 2007, Australia.

[c] Department of Chemistry, University of Otago, Dunedin 9504, New Zealand.

[d] Australian Synchrotron, Australian Nuclear Science and Technology Organisation, New South Wales 2234, Australia.

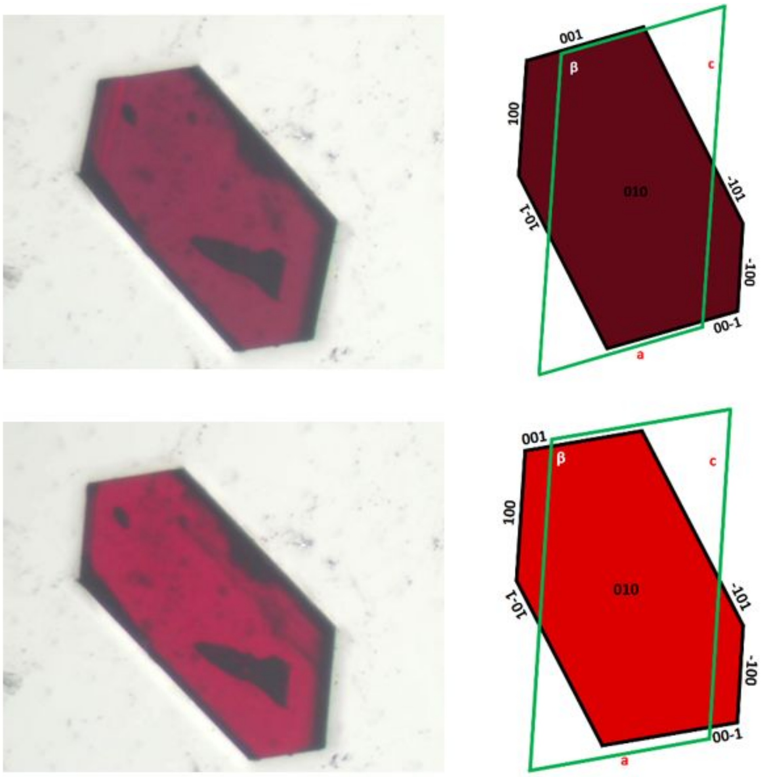

Figure **S1**: Optical photomicrographs, BFDH modelled geometries with face indices and unit cell (in green) overlays for the high temperature (upper) and low temperature (lower) phases of **1** and **2**.

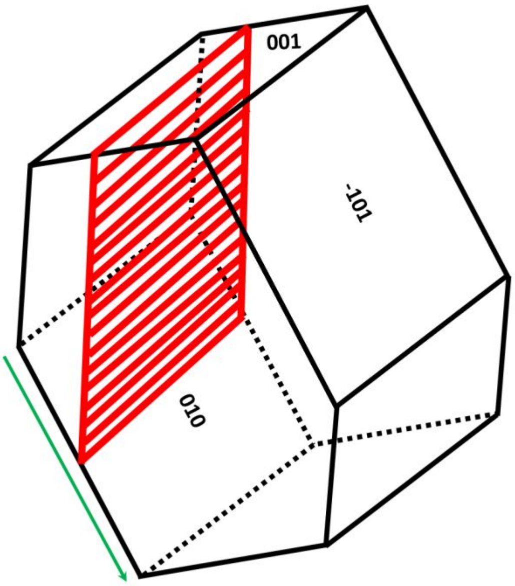

Figure **S2**: Artistic representation of a BFDH modelled single-crystal demonstrating progression (green arrow) of the thermosalient domain wall (shown in red).

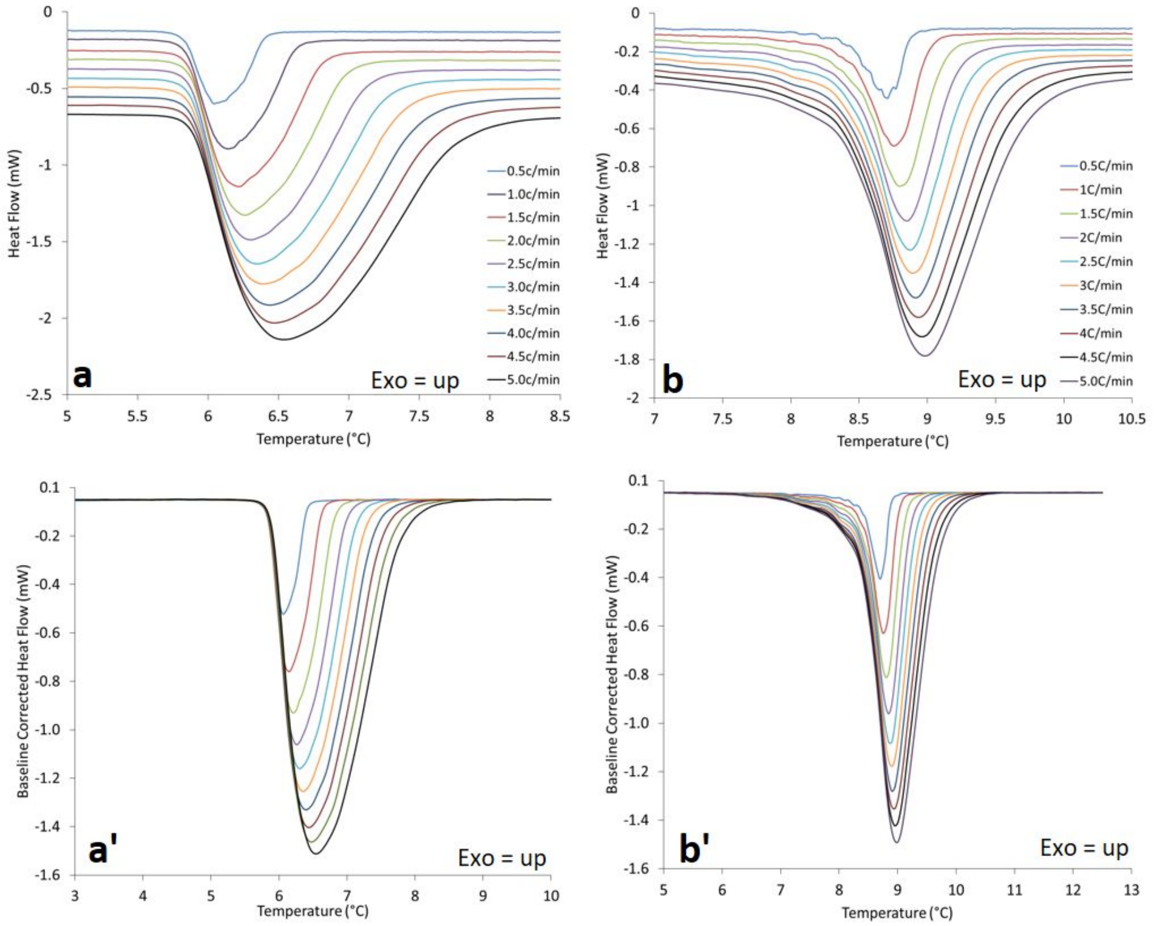

Figure **S3**: Uncorrected endothermic transformation (low temperature to high temperature phase transformation) peaks at variable heating rates for **1** (a) and **2** (b). Baseline corrected endothermic transformation peaks for **1** (a') and **2** (b'). The baseline corrected heat flows were integrated step-wise to produce Figure 3b of the main text.

Details for a supplementary movie of KOALA II diffraction images

The movie “Diffraction spots” is of the same diffraction peaks on heating from the pure LT phase to the pure HT phase. Temperatures were kept constant except for four step increases of 0.2 K, 0.1K, 0.1K, and 0.1K just before frames 2, 13, 19, and 25, respectively. Frame 1 is for the pure LT phase and at 281.75K, frames 2-12 are for 281.95K, frames 13-18 for 282.05K, frames 19-24 for 282.15K, and frames 25-34 for 282.25K. Differentials between the stated temperature and the true sample temperature are expected to be constant and of the order of 1.0K. The images are 5 minute exposures taken at intervals of ~48 minutes.

The image below shows the Miller Indices for spots that have strong intensities in both the HT and LT phases. Pairs of red and blue spots indicate separated spots for the HT and LT phases respectively, while single spots in pink indicate spots where both phases overlap. Spots positions were generated for frame 21 of the movie, in other frames the spots have moved due to the rotation of the crystal which is held to the mount by grease and PTFE tape.

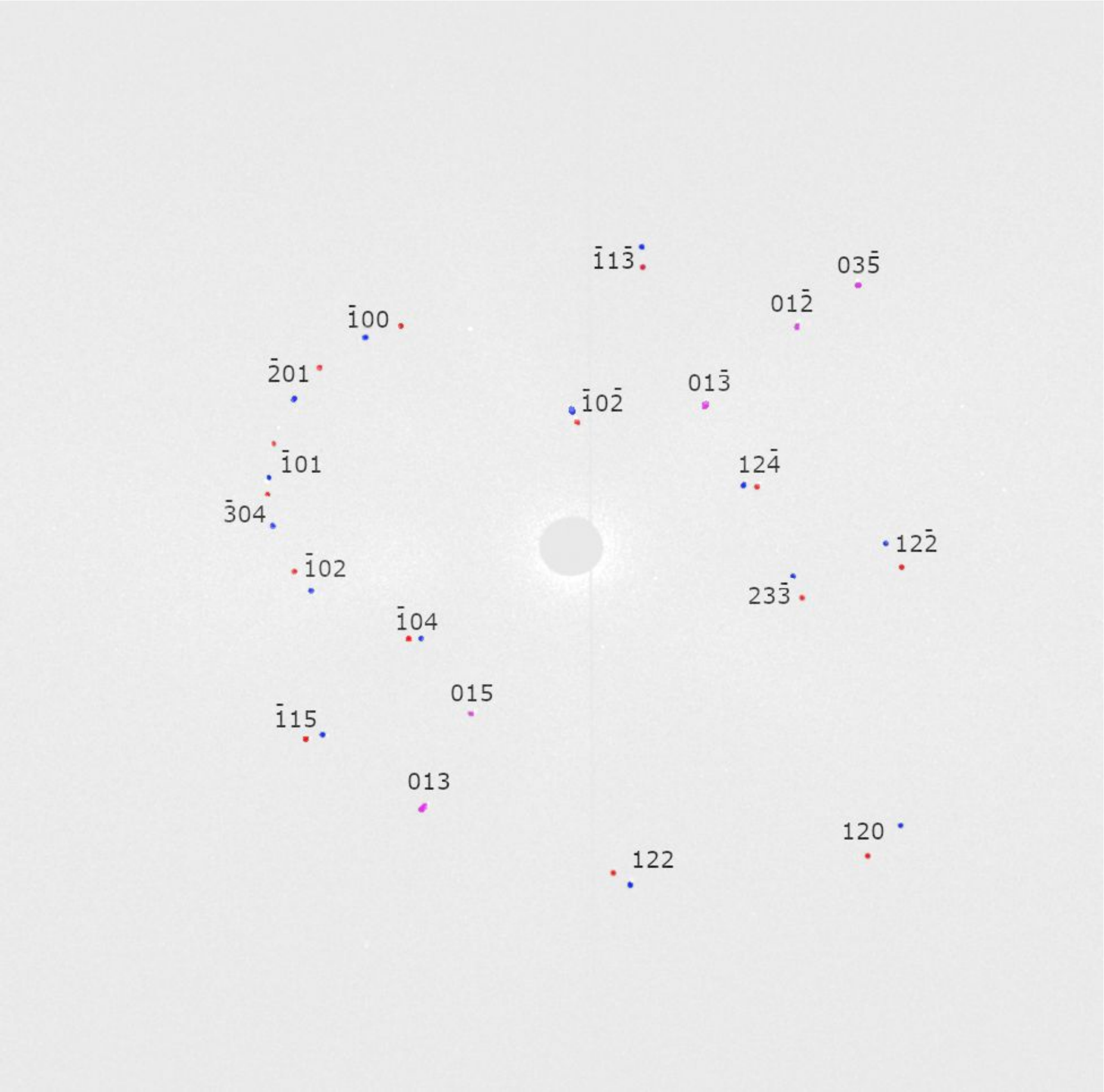

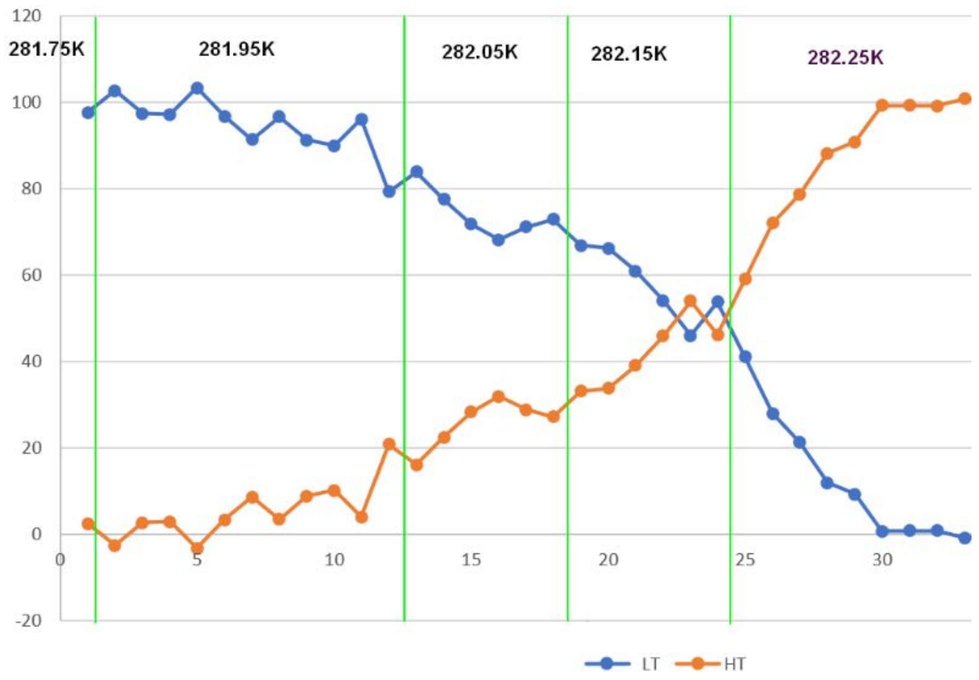

Figure S4: The phase fractions (%) estimated from the intensities of the -3,0,4 spots in the movie.

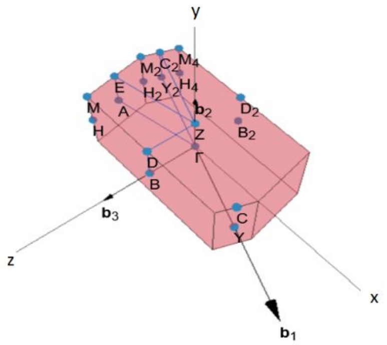

Figure S5: Brillouin wavevectors modelled on the macroscopic crystal. The directions (e.g. D, B, Z) correspond to those as plotted in the phonon dispersion (Figure 7, main text).

Detailed processing methodology for supplementary photomicrography movies

All photomicrograph videos were collected at a fixed frame rate of 10 frames per second. The videos were resized to 1920x1080 resolution to enable efficient processing. The crystal sizes were calibrated using an optical micrometer. Videos were then cropped and saved with JPEG compression to enable upload. For isothermal measurements, the temperature was changed at a rate of 0.1C/min to approach the observation of the thermosalient domain wall (TDW), at which point the temperature was held isothermal. For Thermosalient Domain Walls 1 and 2, the temperatures were calibrated as follows: The temperature at which the TDW is first observed was set to the onset temperature as measured using differential scanning calorimetry at 5 K Min-1 due to limitations on the maximum cooling and heating rate of the calorimeter. The measured temperature on the surface of the substrate at a distance no further than 1.5 mm from the crystal was then used to calibrate the frames using the applied heating or cooling rates. For Thermosalient Domain Wall 1 and 4, the cooling rate was measured at 10.00 +/- 0.1 K min which corresponds to a temperature decrease of 0.016 (rounded to 0.2) K per frame. For Thermosalient Domain Wall 2, the heating rate was 30.00 +/- 0.1 K min which corresponds to a temperature increase of 0.05 K per frame. For Crack, the cooling rate was 80.00 +/- 0.15 K min which corresponds to a temperature decrease of 0.13 K per frame.

Detailed description of supplementary movies

“Crystal Movements” shows the physical movement of large crystals during cooling. Here it can be seen that smaller crystals transition at slightly lower temperatures than large crystals due to their reduced thermal mass.

“Thermosalient Domain Wall 1” shows the progression of the TDW within a single crystal during cooling when viewed down 010. The difference in colors corresponds to changes in birefringence caused by a change in cell beta. The lighter red is the low temperature phase and the darker red is the high temperature phase.

“Thermosalient Domain Wall 2” shows the progression of the TDW within a fragment of a single crystal during cooling. The changes in colour on either side of the thermosalient domain wall are due to changes in birefringence caused by a change in cell β.

“Thermosalient Domain Wall 3” shows the progression of the TDW within a single crystal during cooling. Here, the crystal is sufficiently thick to preclude the observation of birefringence colours thus the high temperature and low temperature phases appear a uniform colour. The TDW is observed due to stress induced changes in the refractive index caused by 'pinning' from the change in cell beta in the adjacent phase.

“Thermosalient Domain Wall 4” shows the progression of the TDW in a single crystal of **2** during cooling. The TDW is characterised as a broad rectangle as the axis of observation is along -101 (See Figure S2).

“Crack” shows the disintegration of a single crystal subjected to a cooling rate of 80 K Min. Here, the TDW originates on both external and internal faces. The crystal cracks due to the large thermal gradient.

“Isothermal” shows the stationary TDW under isothermal conditions on the crystal after cracking. The cracked crystal was chosen by design to demonstrate the resilient behavior of the thermosalient system and TDW even after 'crystal destruction'. Movements in the frames between 1 minute 46 seconds and 1 minute 56 seconds are the result of physical stimulation of the crystal. The slight progression of TDW’s between 2 minutes 30 seconds to 8 minutes are due to minute temperature increases in the ambient environment and correspond to slight increases in temperature as measured by the temperature sensor.

“Imaginary Modes 1-3” are visualisations of atomic displacements of Molecule B in the high temperature phase, corresponding to the phonon dispersion modes with negative energies. These displacements are calculated using self-consistent-field relaxed 211 supercells on which 294 finite atom displacements were applied.

“Diffraction Images” contain the images of diffraction spots as the temperature is increased from fully LT phase to fully HT phase.
